# Prognostic interaction between expression of p53 and estrogen receptor in patients with node-negative breast cancer: results from IBCSG Trials VIII and IX

**DOI:** 10.1186/bcr3348

**Published:** 2012-11-05

**Authors:** Alan S Coates, Ewan KA Millar, Sandra A O'Toole, Timothy J Molloy, Giuseppe Viale, Aron Goldhirsch, Meredith M Regan, Richard D Gelber, Zhuoxin Sun, Monica Castiglione-Gertsch, Barry Gusterson, Elizabeth A Musgrove, Robert L Sutherland

**Affiliations:** 1 The Kinghorn Cancer Centre & Cancer Research Program, Garvan Institute of Medical Research, Darlinghurst, New South Wales 2010, Australia; 2 School of Public Health, University of Sydney, New South Wales 2006, Australia; 3 International Breast Cancer Study Group, Bern, Switzerland; 4 Department of Anatomical Pathology, South Eastern Area Laboratory Service, St George Hospital, Kogarah, New South Wales 2217, Australia; 5 School of Medicine and Health Sciences, University of Western Sydney, Campbelltown, New South Wales 2560, Australia; 6 School of Medical Sciences, Faculty of Medicine, University of NSW, Kensington, New South Wales 2052, Australia; 7 St Vincent's Clinical School, Faculty of Medicine, University of NSW, Kensington, New South Wales 2052, Australia; 8 Department of Tissue Pathology and Diagnostic Oncology, Royal Prince Alfred Hospital, Camperdown, New South Wales 2050, Australia; 9 Central Clinical School, University of Sydney, New South Wales 2006, Australia; 10 International Breast Cancer Study Group Central Pathology Office, Division of Pathology and Laboratory Medicine and European Institute of Oncology, Milan 20141, Italy; 11 University of Milan, Milan 20122, Italy; 12 Division of Medical Oncology, Department of Medicine, European Institute of Oncology, Milan 20141, Italy; 13 IBCSG Statistical Center, Dana Farber Cancer Institute, Harvard Medical School, Harvard School of Public Health Boston, MA 02215, USA

## Abstract

****Introduction**:**

The prognostic significance of p53 protein expression in early breast cancer remains uncertain, with some but not all studies finding an association with poorer outcomes. Estrogen receptor (ER) expression is both a positive prognostic marker and predictive of response to endocrine therapies. The relationship between these biomarkers is unknown.

****Methods**:**

We constructed tissue microarrays (TMAs) from available pathological material from 1113 patients participating in two randomized clinical trials comparing endocrine therapy alone versus chemo-endocrine therapy in node-negative breast cancer. Expression of p53 defined as >10% positive nuclei was analyzed together with prior immunohistochemical assays of ER performed at central pathological review of whole tumor sections.

****Results**:**

ER was present (i.e. >1% positive tumor cell nuclei) in 80.1% (880/1092). p53 expression was significantly more frequent when ER was absent, 125/212 (59%) than when ER was present, 171/880 (19%), p <0.0001. A significant qualitative interaction was observed such that p53 expression was associated with better disease-free survival (DFS) and overall survival (OS) among patients whose tumors did not express ER, but worse DFS and OS among patients whose tumors expressed ER. The interaction remained significant after allowance for pathologic variables, and treatment. Similar effects were seen when luminal and non-luminal intrinsic subtypes were compared.

****Conclusions**:**

Interpretation of the prognostic significance of p53 expression requires knowledge of concurrent expression of ER. The reason for the interaction between p53 and ER is unknown but may reflect qualitatively different p53 mutations underlying the p53 expression in tumors with or without ER expression.

****Trial registration**:**

Current Controlled Trials ACTRN12607000037404 (Trial VIII) and ACTRN12607000029493 (Trial IX).

## Introduction

The p53 tumor suppressor protein, encoded by the *TP53 *gene, is a transcription factor that when activated as part of the cellular stress response, regulates suites of genes involved in cellular processes including the cell cycle, apoptosis, and senescence [1]. Mutations in *TP53 *are amongst the commonest genetic alterations in human cancer, but unlike other tumor suppressors, *TP53 *is not usually inactivated through deletion or truncating mutations [2]. Instead, it commonly undergoes mono-allelic missense mutations affecting the DNA binding domain, leading to the production of a protein that lacks DNA binding activity but remains capable of binding to, and hence dominantly inactivating, wild-type p53. Some *TP53 *mutations also result in the acquisition of new oncogenic properties, and it is unclear whether loss of wild-type function, gain of oncogenic function, or their combined effects, account for the oncogenicity of these mutations [1,2]. Interpretation of many experiments addressing this issue is complicated by the recent identification of multiple p53 isoforms, which arise from alternative splicing and the presence of an internal promoter [3]. The principal isoforms differ in the domain responsible for oligomerization, but are identical through the transactivation and DNA binding domains in which many *TP53 *mutations occur.

Wild-type p53 protein is rapidly degraded, except under conditions of cellular stress. *TP53 *mutations are often, although not always, associated with the production of a stable protein that is readily detectable by immunohistochemistry (IHC) of cancer cells [1,2]. IHC detection of p53 protein is therefore loosely, but imperfectly, associated with mutations in *TP53*. Some studies assessing p53 status using either IHC or mutational analysis have concluded that *TP53 *mutation is associated with poor prognosis, but other authors have reported no impact of *TP53 *mutation on outcome in early breast cancer, and the evidence is not sufficiently strong for p53 status to be recommended as a marker in routine clinical practice [4]. Studies of women treated with a variety of chemotherapeutic regimens, or hormonal therapy suggest that p53 status may be predictive of response to therapy [5,6]. However, until recently few large studies have either considered, or been able to effectively control for, treatment effects. Retrospective analyses of randomized clinical trials, using either IHC measurement of p53 expression (CALB 9344) or *TP53 *gene sequencing (BIG 02-98), have identified a significant association with a worse prognosis in patients treated with adjuvant doxorubicin and cyclophosphamide or doxorubin but no significant association with response to taxanes [7,8]. Similarly, a prospective clinical trial (EORTC 10994/BIG 1-00) found that *TP53 *status was not predictive of response to taxane-based therapy [9].

The different methods used to assess p53 status have both advantages and disadvantages. Direct identification of mutations by sequencing has the advantage of pinpointing the specific aberration, but is not currently applicable to clinical practice because of its expense, technical difficulty, and likely reduced sensitivity in the routine setting. In addition, it may mis-classify some functionally equivalent genomic alterations, where *TP53 *is inactivated indirectly. Functional assessment of p53 activity in yeast offers a distinction between inactivating and other mutations [10,11]. Several laboratories have attempted to develop gene signatures of p53 inactivation, which can potentially be measured using a PCR-based test [12-14]. However, these assays are not immediately practical for routine diagnosis, and some of these signatures are potentially measures of subtype rather than p53 status, since mutations in *TP53 *are more frequent in tumors lacking estrogen receptor (ER) expression [15-17], in tumors of basal-like, HER2 and luminal B subtypes [18,19], tumors showing stem-cell like transcriptional patterns [20] and in those with high proliferative fraction [21]. IHC detection of p53 protein mis-classifies some *TP53 *mutations, and instances where *TP53 *is inactivated through deletion or truncation, or where persistent cellular stress leads to sustained expression of p53 protein. However, it does have the advantage of ready transfer into clinical practice, if studies in large, well-characterized cohorts provide good evidence of potential utility as a biomarker. Since the material available for the present study was limited to tissue micro-arrays, IHC was the most suitable available method.

Evidence for cross-talk between ER and p53 pathways at several levels suggests that the impact of *TP53 *mutation may be affected by the presence of ER. First, ER is a p53 target and conversely estrogen treatment induces p53 expression, although it has also been reported to increase the cytoplasmic localization of p53, thereby functionally inactivating it [22-24]. Second, p53 is required for hormonal protection against carcinogen-induced mammary cancer in rodents [25,26]. Finally, the ER and p53 proteins physically interact, leading to repression of their transcriptional activity and protection of p53 from degradation [27-29]. However, other authors have concluded that the dominant interaction is via ER and p53 binding their cognate response elements *in cis *to cooperatively regulate p53-responsive genes [30]. Simultaneous allowance for p53 and ER in survival analysis of patients with early breast cancer, where the presence of ER is a favorable prognostic factor [31-34], and predicts the efficacy of endocrine therapies [31], has yielded conflicting results. Some authors report independent adverse prognostic significance of ER-negativity and p53 expression [15,35] while more recently, different p53 gene signatures have been associated with disparate prognosis and response to cytotoxic therapy in ER-positive and ER-negative disease [12].

As a step towards addressing some of these issues, we have explored the relationship between ER expression and p53 expression detected by immunohistochemistry on prognosis in the context of large prospective randomized clinical trials. In the present study we have examined available pathological material from International Breast Cancer Study Group (IBCSG) Trials VIII and IX, comparing endocrine adjuvant therapy alone versus combined modality chemo-endocrine therapy in patients with node-negative early breast cancer. This revealed a qualitative interaction between ER and p53 expression, such that p53 expression was prognostically adverse in patients whose tumors expressed ER, but favourable in those whose tumors lacked ER expression.

## Materials and methods

### Trials and patients

The designs of IBCSG Trials VIII and IX have been described in detail elsewhere [36,37]. Briefly, from 1990 to 1999 in Trial VIII, 1,063 pre- and peri-menopausal women with node-negative early breast cancer were randomly assigned to endocrine therapy with 24 months of goserelin alone, six cycles of chemotherapy with classical cyclophosphamide, methotrexate and 5-fluorouracil (CMF) or a sequence of 6 cycles of CMF followed by 18 months of goserelin. Similarly, from 1988 to 1999 in Trial IX, 1,669 eligible postmenopausal women were randomly assigned endocrine therapy with 5 years of tamoxifen 20 mg daily or three cycles of CMF followed by tamoxifen to complete 5 years therapy. In each trial, randomization was stratified according to locally-determined ER status. Institutional review boards reviewed and approved the protocols, and informed consent was required according to the criteria established within the individual countries. Patient follow-up, vital status and date of any relapse or recurrence are recorded in the IBCSG database. Median follow-up from randomization in Trial VIII is 12 years [36] and in Trial IX is 13 years [37]. IBCSG Trials VIII and IX were conducted from 1988 to 1999. Ethical approval was obtained in participating countries according to national regulations. The IBCSG Independent Data Monitoring Committee reviewed the trials periodically. All patients included in the analysis provided consent to participate in the trials. The study was reviewed and approved by the IBCSG Biological Protocols Working Group.

### Pre-existing pathological data

Central review of immunohistochemical expression of ER in whole sections has been documented [38,39]. The data presented in these published reports is incorporated in a cumulative IBCSG database, which has been used to classify ER status in the present study. Similarly, the central review assessment of tumor size, Bloom and Richardson grade [39], and peri-tumoral vascular invasion, as previously described [40], were used for the present study. HER2 was considered as positive if 3+ by IHC at central assessment or amplified by fluorescent *in situ *hybridisation (FISH) performed on the tissue microarrays (TMAs) (HER2: C17 ratio > 2.0). There is debate about the threshold at which tumors should be considered positive for ER [41,42]. Following IBCSG practice [39] and the recommendations of the American Society of Clinical Oncology and the College of American Pathologists [43], and before any data analysis, we defined a cut-point to identify ER as present if at least 1% of cells showed ER staining on immunohistochemistry.

### Tissue microarrays

**Figure 1:**
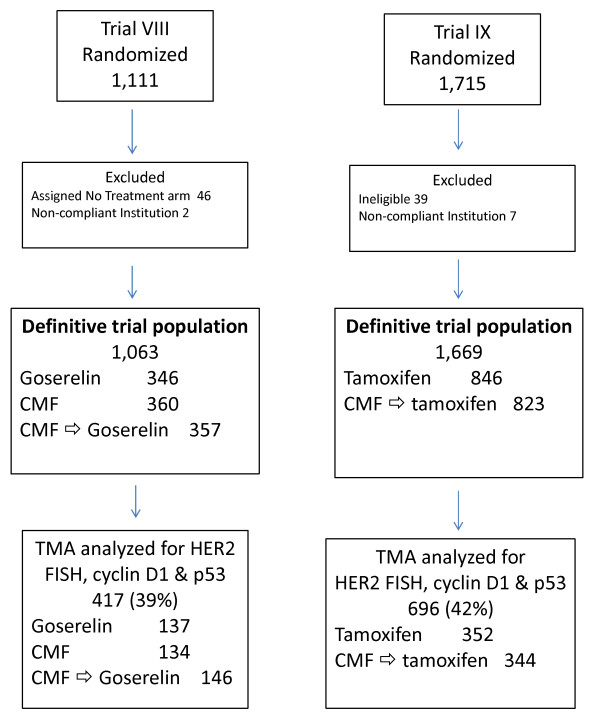
**Remark diagram showing patients randomized to the parent clinical trials and those included in the present cohort for analysis of p53 expression**. CMF, cyclophosphamide, methotrexate, 5-fluorouracil; FISH, fluorescent *in situ *hybridisation; HER, human epidermal growth factor receptor 2.

Available tissue blocks from 1,493 patients randomized to IBCSG Trials VIII (593 patients) and IX (900 patients) were sent from the IBCSG Pathology Office to the Cancer Research Program, Garvan Institute of Medical Research, Sydney, Australia for construction of tissue TMAs. The TMAs were produced using the MTA-1 Manual Tissue Arrayer and a 1.0 mm needle to biopsy tumor tissue identified by examination of hematoxylin and eosin (H&E)-stained slides from a standard histological block. Three representative cores were taken from each donor block and deposited in the recipient array block. Each array in Trial VIII comprised 108 (9 ± 12) cores representing about 32 patients. Arrays from Trial IX comprised 96 cores (8 ± 12) representing about 28 patients. An asymmetric template was employed for core orientation. Cores of renal tissue (± 6) were randomly placed within each array to act as orientation markers when scoring. Normal breast cores taken from reduction mammoplasties (± 6) were also placed on each array to allow comparison between immunohistochemical staining in morphological normal breast and invasive breast carcinoma. Details of numbers of patients randomized and those analyzed in the present study are given in the REMARK Diagram (Figure 1) and in Table 1. Briefly, after exclusion of ineligible patients and those from non-compliant institutions, TMAs were prepared from 1,220 patients (Trial VIII, 450; Trial IX, 770). Staining for p53 as described below was successfully performed on TMAs from 1,113 patients (Trial VIII, 417; Trial IX, 696). Comparison of patients assessed for p53 and those not so assessed is presented in Table 1. Centrally reviewed ER was unavailable for 21 patients (Trial VIII, 11; Trial IX, 10) leaving a study cohort for the present analysis of 1,092 patients (Trial VIII, 406; Trial IX, 686).

**Table 1:** Patient characteristics by analysis cohort

	Trial VIII		Trial IX		Pooled Trials	
p53	Analyzed	Not analyzed	Analyzed	Not analyzed	Analyzed	Not analyzed
Number of patients	417	646	696	973	1,113	1,619
Median age (range)	46	45	61	60		
	(26 to 58)	(22 to 56)	(42 to 77)	(34 to 81)		
		ns		ns		
ER status, n						
Absent	84	101	128	137	212	238
> = 1%	322	360	558	516	880	876
Missing	11	185	10	320	21	505
		ns^1^		ns		
Tumor size, n						
0 to 1 cm	27	99	65	136	92	235
> 1 to 2 cm	206	321	333	450	539	771
> 2 cm	182	217	280	352	462	569
Missing	2	9	18	35	20	44
		*P *< 0.0001		*P *= 0.01		
Tumor grade, n						
	52	94	85	160	137	254
1	171	286	304	447	475	733
2	168	179	248	261	416	440
3	26	87	59	105	85	192
Missing		P = 0.002		*P *= 0.0004		
10-year DFS	71.3%	76.0% *P *= 0.15^2^	67.6%	75.5% *P *= 0.10		
10-year OS	86.4%	87.4% *P *= 0.44	81.1%	83.8% *P *= 0.65		

^1^*P*-values compare distribution of non-missing values between analyzed and non-analyzed cohorts; ns, not significant. ^2^DFS and OS *P*-values based on logrank comparison of entire curves. n, number of patients; DFS, disease-free survival; OS, overall survival.

### TMA staining

Four-μm sections were baked in an oven at 63°C for 2 hours followed by rehydration in graded ethanols, followed by water. Antigen retrieval was performed by boiling in a water bath for 30 minutes at pH 9.0 using Dako antigen retrieval solution (S2367, Dako, Denmark). All additional steps were performed on a Dako autostainer: p53 monoclonal antibody (clone DO-7 Dako, Denmark) was incubated at 1:200 dilution for 30 minutes at room temperature, following standard blocking procedures with 3% hydrogen peroxide for 5 minutes. Detection involved Envision labeled polymer-horseradish peroxidise (HRP) anti-mouse antibody (Dako, Denmark) which was added for 30 minutes at room temperature, followed by diaminobenzidine (DAB)+ chromogen (Dako, Denmark) for 10 minutes. Slides were then counterstained with haematoxylin, dehydrated and mounted. Isotype-matched non-specific immunoglobulin was substituted for the primary antibody as the negative control. A p53 positive breast carcinoma was used as the positive control.

### P53 scoring

All scoring was performed by an experienced breast pathologist blinded to all clinical and outcome information. Maximum nuclear staining > 10% (any intensity) was considered positive, as employed in most studies using this antigen. Data from the scoring assessment of p53 on the TMAs were entered into, the data handling program (Cansto) at the Garvan Institute of Medical Research, Sydney.

### Statistical analysis

Data on baseline characteristics and follow-up were extracted from the IBCSG clinical and pathological database and merged with the p53 data obtained from TMAs. The trial endpoint was disease-free survival (DFS), defined as the length of time from the date of randomization to any invasive breast cancer relapse (including ipsilateral or contralateral breast recurrence), the appearance of a second non-breast malignancy, or death, whichever occurred first, or was censored at the date of last follow-up. Overall survival (OS) was defined as the length of time from randomization to death from any cause, or was censored when last known alive. OS and DFS were estimated using the Kaplan-Meier method and compared using the logrank test. The association of covariates and of interactions between variables with OS and DFS was assessed using proportional hazard models. Since similar effects and interactions were observed separately for each trial (data not shown) the definitive analyses included all available patients. Multivariate DFS and OS analyses were stratified by trial, using SAS software version 9.2. Proportional hazards assumptions were checked using martingale residuals. All *P*-values are two-sided.

## Results

### Characteristics of analyzed patients compared to other trial participants

Blocks available for this study were those with residual tumor after routine pathology, central review and the conducting of previous translational research studies using whole tumor sections [39,44,45]. Thus the 1,113 patients with p53 results available for this project were more likely to be those with more advanced tumors than those not included. This is reflected in the significantly higher proportion in larger primary tumor size categories and with higher Bloom and Richardson grade in patients available from both trials. There was no significant difference in age distribution or the presence of ER and the trends to worse DFS and OS were not statistically significant (Table 1).

### Impact of p53 status and ER, and their interaction on outcome

**Figure 2:**
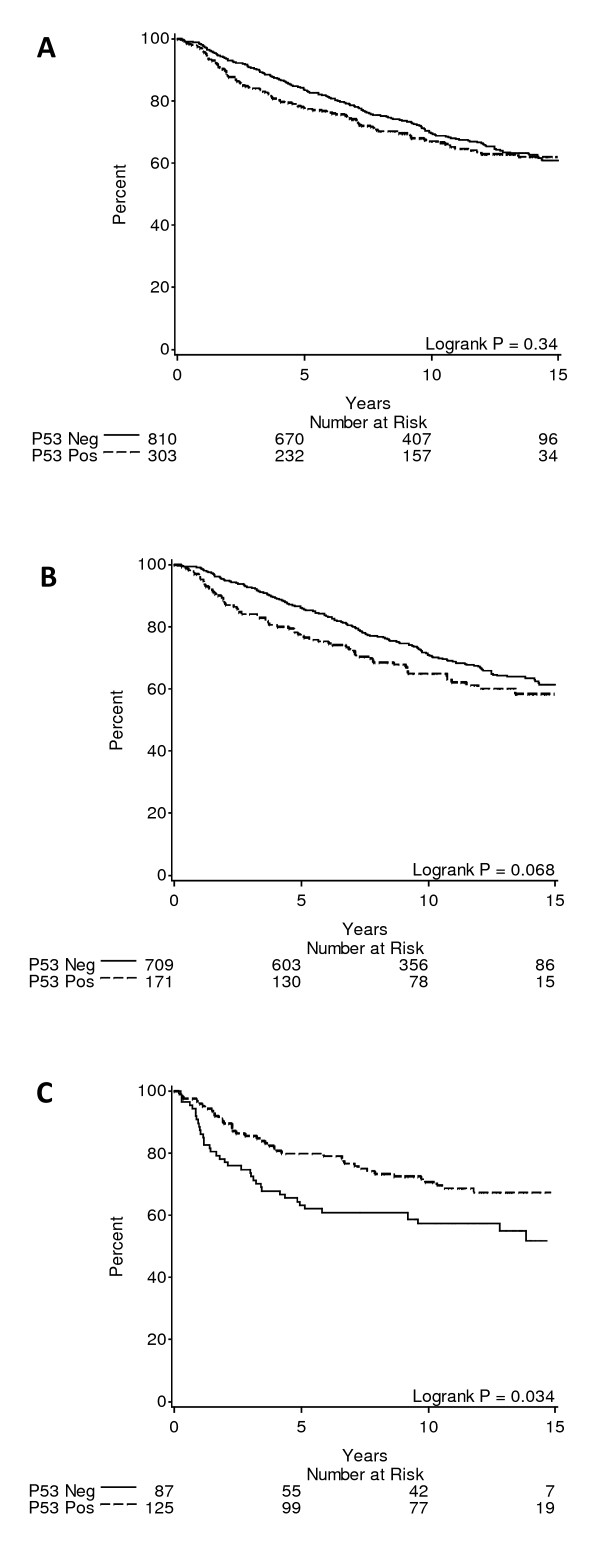
**Disease-free survival by p53 expression (≥ 10%) at 12-year median follow-up**. (**A**) All analysed patients. (**B**) Patients whose tumors expressed estrogen receptor (ER). (**C**) Patients whose tumors did not express ER.

**Figure 3:**
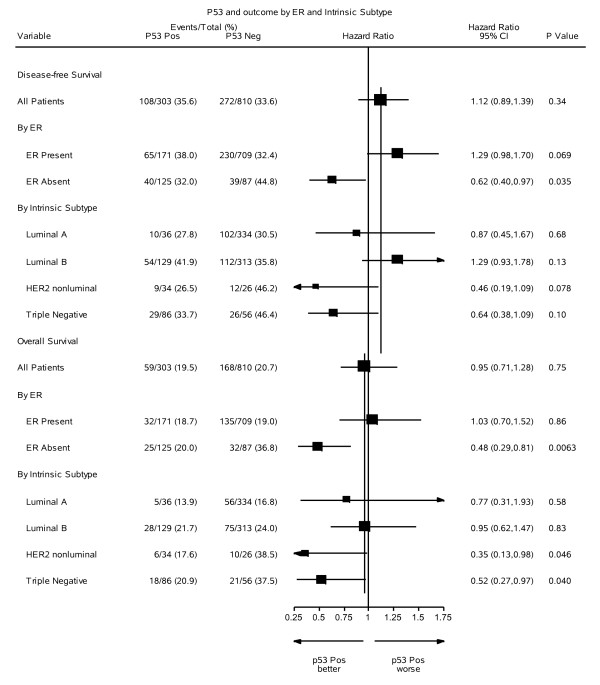
**Forest plots of hazard ratios for disease-free and overall survival by p53, estrogen receptor (ER) expression and intrinsic subtype**.

Data on p53 status were available in 1,113 patients and for centrally reviewed ER status in 1,092 of these. P53 positivity was more common among patients with absent ER expression (125/212, 59%) than among those expressing ER (171/880, 19%, *P *< 0.0001). In univariate analyses the overall slight negative impact of p53 expression on DFS was not significant (Figure 2A). In the presence of ER expression, the adverse effect of p53 staining was more marked while in the absence of ER, p53 expression was associated with better DFS (Figure 2B and 3).

A statistically significant interaction was present between p53 status and ER (*P *= 0.004), as shown in Table 2. The statistical significance of the interaction persisted for both DFS and OS in further models allowing for significant pathological variables, and for treatment allocation and its interaction with ER status (Table 2). We found no evidence of interaction between p53 expression and the relative efficacy of the various endocrine or chemo-endocrine therapies used in the trials (data not shown), indicating that in our studies p53 expression was not a predictive marker for the efficacy of adding CMF chemotherapy to endocrine adjuvant therapy. Martingale residuals reflected no violations of proportionality apart from the ER variable. Tumors lacking ER are known to exhibit higher initial failure rates [46].

**Table 2:** Interaction between presence of estrogen receptor (ER) and p53 expression

	Hazard ratio	95% Confidence interval	*P*-value
Disease-free survival models^1^			
p53 univariate (positive vs negative)	1.128	0.902, 1.410	0.3
ER univariate (present vs absent)	0.850	0.663, 1.090	0.2
Without interaction			
p53	1.044	0.815, 1.339	0.7
ER	0.866	0.660, 1.138	0.3
With interaction			
p53	0.606	0.390, 0.942	0.03
ER	0.606	0.431, 0.851	0.004
Inter p53/ER^2^	2.152	1.278, 3.623	0.004
With pathological variables^3^			
p53	0.575	0.368, 0.896	0.01
ER	0.747	0.517, 1.081	0.12
Inter p53/ER	1.968	1.156, 3.352	0.01
Grade 1	1.00	Reference	0.0009^4^
Grade 2	1.463	1.018, 2.104	
Grade 3	1.988	1.362, 2.903	
Vascular invasion^5^	1.314	1.040, 1.661	0.02
With pathological and treatment variables			
p53	0.581	0.373, 0.906	0.02
ER	0.957	0.608, 1.507	0.85
Inter p53/ER	1.941	1.139, 3.307	0.01
Grade 1	1.00	Reference	0.0009
Grade 2	1.458	1.014, 2.097	
Grade 3	1.988	1.361, 2.904	
Vascular Invasion	1.305	1.033, 1.650	0.03
Tmt Endocrine only^6^	1.688	1.081, 2.636	0.02
Inter Tmt/ER^7^	0.595	0.360, 0.984	0.04
OS models			
p53 univariate	0.985	0.731, 1.326	0.9
ER univariate	0.667	0.493, 0.901	0.008
Without interaction			
p53	0.783	0.561, 1.092	0.15
ER	0.599	0.430, 0.836	0.003
With interaction			
p53	0.460	0.273, 0.777	0.004
ER	0.434	0.295, 0.638	<0.0001
Inter p53/ER	2.350	1.225, 4.507	0.01
With pathological variables			
p53	0.426	0.252, 0.719	0.001
ER	0.498	0.325, 0.763	0.001
Inter p53/ER	2.293	1.187, 4.433	0.01
T < = 1 cm	1.00	Reference	0.003
T >1 to 2 cm	1.858	0.932, 3.706	
T > 2 cm	2.682	1.340, 5.368	
Grade 1	1.00	Reference	0.11
Grade 2	1.578	0.957, 2.604	
Grade 3	1.759	1.041, 2.973	
Vascular invasion	1.309	0.971, 1.764	0.08
With pathological and treatment variables			
p53	0.427	0.253, 0.723	0.002
ER	0.604	0.355, 1.028	0.06
Inter p53/ER	2.280	1.179, 4.409	0.01
T < = 1 cm	1.00	Reference	0.002
T > 1 to 2cm	1.929	0.965, 3.856	
T > 2 cm	2.762	1.378, 5.536	
Grade 1	1.00	Reference	0.11
Grade 2	1.573	0.954, 2.594	
Grade 3	1.749	1.034, 2.957	
Vascular invasion	1.295	0.961, 1.745	0.09
Tmt endocrine only	1.574	0.931, 2.659	0.09
Inter Tmt/ER	0.666	0.363, 1.222	0.19

^1^Stratified by trial. ^2^Interaction term reflects cases with both expression of p53 and presence of ER. ^3^Tumor size was not significant and was dropped from the disease-free survival model. ^4^Peritumoral vascular invasion at central pathology review. ^5^*P*-values for multi-level variables (tumor size and grade) reflect overall significance. ^6^Treatment allocation endocrine only (goserelin in trial VIII, tamoxifen in trial IX) without chemotherapy. ^7^No interaction was seen between p53 status and the presence or absence of chemotherapy (data not shown). Inter, interaction (as detected by statistical analysis); Tmt, treatment allocation.

The classification of ER presence (if at least 1% of tumor cells showed ER staining on IHC) was defined prior to any data analysis. Exploration of an alternative ER-positive cut-point based on staining of at least 10% of cells [41], resulted in reclassification of 45 patients with ER staining levels of 1 to 9%, and this materially reduced the clarity of the interaction with p53 status (DFS interaction hazard ratio (HR) 1.76, *P *= 0.04, OS interaction HR 1.68, *P *= 0.11).

Reflecting the effect of ER expression, a similar dichotomy of impact of p53 expression was seen when patients were divided into luminal and non-luminal subtypes based on IHC expression of ER, progesterone receptor and Ki67, as well as the detection of HER2 by IHC (3+) or FISH [47] (Figure 3).

## Discussion

The present study is unusual in demonstrating a significant qualitative interaction between two biological markers. Detectable p53 expression was associated with better prognosis in patients whose tumors did not express ER but with worse prognosis if ER was expressed. This interaction appeared robust in the two trials examined and was independent of other pathologic features and of treatment. However, this and the similar interaction between p53 status and intrinsic subtype (as assessed by IHC) were unexpected and therefore require confirmation in large independent data sets for which p53 status is known to be available, such as that recently described by Lara *et al. *for the Cancer and Leukemia Group B [7], the EORTC 10994/BIG 1-00 trial described by Bonnefoi *et al. *[9], or the BIG 2-98 trial described by Francis *et al. *[48].

If confirmed, such an interaction might at least partially explain the earlier disparate reports of the prognostic significance of p53 staining viewed in isolation. Based on our exploratory analysis of a higher ER cut-point we suggest that any confirmatory study should look primarily at ER-present (IHC > 1% staining) versus ER-absent staining. Our observation that the interaction is most clearly seen with an ER cut-point reflecting ER expression versus absence of expression may explain the failure to observe such an interaction in earlier small studies which typically used a higher ER cut-point [15,16,35].

If the interaction between ER and p53 as markers of prognosis is biologically real, its basis is currently unclear, although there is evidence for functional relationships between p53 and ER that affect mammary oncogenesis and/or response to tamoxifen. In genetically engineered mice, *p53 *heterozygosity leads to increased mammary epithelial proliferation, decreased apoptosis and eventual development of pre-neoplastic mammary lesions [49]. These responses are all enhanced in the presence of deregulated ER expression [49]. A direct interaction between p53 and ER has been described to inhibit p53-responsive transcriptional activation in mammospheres, but this response is antagonised by tamoxifen, suggesting a mechanism that could contribute to a better response to tamoxifen in women with wild-type p53 [50]. Our observation that p53 positivity is associated with a worse prognosis in women treated with tamoxifen in the context of a randomised clinical trial is consistent with this idea.

*TP53 *mutations are not only more common among breast cancers not expressing ER, and among the basal-like and HER2 molecular subtypes, which typically lack ER expression [18] but also tend to be different in type [12]. Mutations are non-randomly distributed along the p53 domains [5,51]. Different p53 mutations have been described as carrying differing adverse prognostic significance [5,8,17]. It is possible that p53 staining by IHC seen in tumors not expressing ER reflects mutations that are not disabling, or are less disabling, to cell homeostasis than those responsible for p53 IHC staining in tumors expressing ER.

As well as the well-studied canonical p53 protein, normal breast tissue expresses the p53β and p53γ isoforms, which arise from alternative splicing and differ at the carboxy-terminus [3]. Most *TP53 *mutations will result in changes in all three isoforms, and there are as yet no reagents for IHC that distinguish between them. However, different patterns of isoform expression are apparent in breast cancer: p53β mRNA expression is associated with ER expression, while p53γ mRNA expression is associated with *TP53 *mutation [52]. Each has been detected by PCR in 36 to 37% of breast cancers, but only 19% were found to express both [52]. Since the antibody used in this study recognises all three isoforms and the p53β protein isoform is more stable than the other isoforms [53], it is possible that p53 IHC positivity represents a different spectrum of isoforms in cancers expressing, or not expressing ER. The isoforms are differently regulated and may have different functions [3]. In particular, the physical interaction between ER and p53 occurs via the carboxy-terminal domain, which differs in sequence between the different p53 isoforms [29]. Although patients expressing mutant p53 but not p53γ have been shown to have a particularly poor prognosis, those expressing mutant p53 and p53γ have a good prognosis, indistinguishable from patients expressing wild-type p53 [52]. This complexity in the prognostic impact of *TP53 *mutations may contribute to the interaction with ER observed here.

Future studies should attempt to clarify this relationship by use of material from patients with p53 staining to ascertain the nature of the p53 mutations involved and examine their prognostic significance. Meanwhile it seems prudent to encourage independent validation of the current data in other large randomized clinical trials and to interpret the prognostic significance of IHC detection of p53 in the context of ER expression.

## Conclusions

IHC detection of p53 protein in early breast cancer loosely reflects the presence of p53 mutations but its prognostic significance is inconsistently reported. In patients with node-negative breast cancer who participated in International Breast Cancer Study Group Trials VIII and IX we show that the relationship between p53 expression and DFS and OS was dependent on ER status. Among patients whose tumors expressed ER, p53 expression was associated with inferior DFS and OS, whereas p53 expression was associated with better DFS and OS in patients whose tumors did not express ER. The interaction was statistically significant, and remained so in models including other pathological variables. Similarly, p53 was associated with worse prognosis among patients with luminal tumor subtypes but better prognosis among those with triple-negative or HER2-positive subtypes. Interpretation of the prognostic significance of p53 staining requires consideration of ER status.

## Abbreviations

CALGB: Cancer and Leukemia Group B; CMF: cyclophosphamide, methotrexate, 5-fluorouracil; DAB: diaminobenzidine; DFS: disease-free survival; EORTC: European Organisation for the Research and Treatment of Cancer; ER: estrogen receptor; FISH: fluorescent *in situ *hybridisation; H&E: haematoxylin and eosin; HER2: human epidermal growth factor receptor 2; HRP: horseradish peroxidise; IBCSG: International Breast Cancer Study Group; IHC: immunohistochemistry; OS: overall survival; PCR: polymerase chain reaction; TMA: tissue microarray.

## Competing interests

The authors declare that they have no competing interests.

## Authors' contributions

ASC, EKAM, SO'T, EAM and RLS participated in the concept and design of the study. ASC, GV, AG, MC, RDG and BG provided materials for the study. ASC, EKAM, SO'T, TJM, EAM, RLS, MMR and ZS performed data analysis and interpretation for the study. ASC, EKAM, SO'T, EAM and RLS drafted, revised and edited the manuscript. All authors read and approved the final draft of the manuscript.
